# Value of diffusion weighted MR imaging as an early surrogate parameter for evaluation of tumor response to high-dose-rate brachytherapy of colorectal liver metastases

**DOI:** 10.1186/1748-717X-6-43

**Published:** 2011-04-27

**Authors:** Christian Wybranski, Martin Zeile, David Löwenthal, Frank Fischbach, Maciej Pech, Friedrich-Wilhelm Röhl, Günther Gademann, Jens Ricke, Oliver Dudeck

**Affiliations:** 1 Department of Radiology and Nuclear Medicine, Otto-von-Guericke University Magdeburg, Germany; 2 Institute of Biometry and Medical Informatics, Otto-von-Guericke University Magdeburg, Germany; 3 Department of Radiotherapy, Otto-von-Guericke University Magdeburg, Germany

## Abstract

**Background:**

To assess the value of diffusion weighted imaging (DWI) as an early surrogate parameter for treatment response of colorectal liver metastases to image-guided single-fraction ^192^Ir-high-dose-rate brachytherapy (HDR-BT).

**Methods:**

Thirty patients with a total of 43 metastases underwent CT- or MRI-guided HDR-BT. In 13 of these patients a total of 15 additional lesions were identified, which were not treated at the initial session and served for comparison. Magnetic resonance imaging (MRI) including breathhold echoplanar DWI sequences was performed prior to therapy (baseline MRI), 2 days after HDR-BT (early MRI) as well as after 3 months (follow-up MRI). Tumor volume (TV) and intratumoral apparent diffusion coefficient (ADC) were measured independently by two radiologists. Statistical analysis was performed using univariate comparison, ANOVA and paired t test as well as Pearson's correlation.

**Results:**

At early MRI no changes of TV and ADC were found for non-treated colorectal liver metastases. In contrast, mean TV of liver lesions treated with HDR-BT increased by 8.8% (*p *= 0.054) while mean tumor ADC decreased significantly by 11.4% (*p *< 0.001). At follow-up MRI mean TV of non-treated metastases increased by 50.8% (*p *= 0.027) without significant change of mean ADC values. In contrast, mean TV of treated lesions decreased by 47.0% (*p *= 0.026) while the mean ADC increased inversely by 28.6% compared to baseline values (*p *< 0.001; Pearson's correlation coefficient of r = -0.257; p < 0.001).

**Conclusions:**

DWI is a promising imaging biomarker for early prediction of tumor response in patients with colorectal liver metastases treated with HDR-BT, yet the optimal interval between therapy and early follow-up needs to be elucidated.

## Background

The liver with its dual blood supply is a predisposed organ for metastatic disease [1]. Colorectal carcinoma (CRC) represents the most frequent malignancy with isolated hepatic metastases [2]. Hepatic resection has become the standard of care and has been shown to lead to a significant improvement of long-term survival, however curative resection is possible in less than 25% of the patients with isolated hepatic metastases [3]. For unresectable metastases selective internal radiation therapy (SIRT) and radiofrequency ablation (RFA) have been shown to be efficient treatment alternatives [4,5].

Image-guided single-fraction ^192^Ir-high-dose-rate brachytherapy (HDR-BT) is a high precision percutaneous ablation technique which has been shown to yield promising results with regards to safety and efficacy in the treatment of unresectable colorectal liver metastases [6-8]. Precise application of high irradiation doses to tumor tissue with steep dose gradients resulting in sparing of adjacent liver parenchyma allows this technique to be applied repeatedly for treatment of recurrent hepatic metastases [9,10]. Nonetheless, it would be of great benefit to be able to evaluate treatment response as early as possible. This would be particularly important in individual cases in which irradiation doses have to be reduced because of diminished functional hepatic reserve or adjacent organs at risk such as stomach or intestine [11]. Early response evaluation in such patients would be of major clinical significance to allow for prompt modification of anticancer treatment, e.g. repeated HDR-BT or additional radiofrequency ablation in underdosed regions, and avoid unnecessary treatment delays.

Diffusion-weighted imaging (DWI) supplies information of water proton mobility [12,13]. This can be employed to assess the microstructural organization of a tissue like cell density, cell membrane integrity and ultimately cell viability which affect water diffusion properties in the extracellular space [14]. Liver DW MR imaging has in the past been hampered by technical challenges, mostly related to motion sensitivity and eddy currents [15]. However, owing to improvement, the technique has also successfully been used in the liver to predict and monitor a variety of anticancer therapies [16-21]. The purpose of this study was to test the hypothesis that DWI can predict tumor response in patients with colorectal liver metastases as early as 2 days after interstitial HDR-BT.

## Methods

### Patient population

The study was approved by the local institutional review board and written informed consent was obtained from each patient. 30 patients (14 women and 16 men; mean age 65.6 years; range: 43 - 84 years) with a total of 43 unresectable colorectal metastases underwent HDR-BT in a total of 37 sessions. Sixteen patients were found surgically unresectable due to unfavourable anatomic localization (bilobar metastases, infiltration of liver vessels), 10 patients had limited extrahepatic disease, and 4 patients presented with comorbidities which excluded resection.

Seven patients underwent previous liver surgery, 25 patients were previously treated with chemotherapy, and two patients received adjuvant chemotherapy within the follow-up period. The follow-up MRI data of these two patients was excluded from analysis. In 13 of these patients, who presented with more than one colorectal liver metastasis, a total of additional 15 lesions were identified which were not treated at the initial session (mean time interval between HDR-BT sessions: 40 days; range: 26 - 66 days). In order to minimize the risk of hepatic toxicity patients with multiple metastases were treated in sequential HDR-BT sessions. These 15 lesions served as control in order to compare changes in tumor volume (TV) and apparent diffusion coefficient (ADC) between treated and non-treated colorectal liver metastases. Patients with tumor diameters less than 1 cm, or poor image quality, e.g. respiratory motion or pulsation artifacts, in which valid quantification of the mean ADC was questionable were excluded from the study.

### Image-Guided Interstitial HDR Brachytherapy

**Figure 1:**
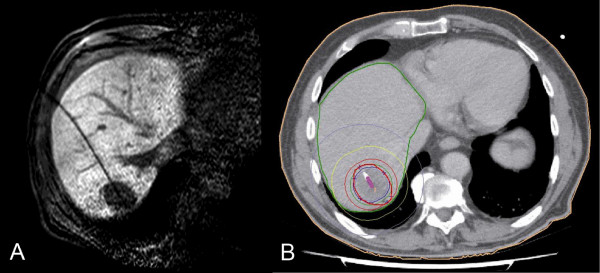
**Illustration of MR-guided HDR-BT and 3D dosimetry**. 77-year-old man with colorectal liver metastasis in segment VII scheduled for high-dose-rate brachytherapy (HDR-BT). The implantation of one brachytherapy catheter was performed under MRI guidance (A). The tumor enclosing dose (D_100_) was 21.8 Gy (B).

Brachytherapy catheters were positioned in analgosedation using either CT fluoroscopy (n = 20; Aqilion 16, Toshiba medical systems, Otawara, Tochigi, Japan) or high field open MRI guidance (n = 23; Panorama, Philips Healthcare, Best, the Netherlands) based on conspicuity of the metastases in either imaging modality. Patients received 0.1 ml/kg body weight of a 0.25 mol/L solution of Gd-EOB-DTPA (Primovist, BayerSchering, Berlin, Germany) prior to MRI guided catheter placement to improve tumor visualization, for which a T_1_-weighted gradient echo sequence (T1 FFE; TR = 11 ms, TE = 6 ms, flip angle = 35°, section thickness of 8 mm, image acquisition every 1.1 s) was used. For adequate coverage of the target volume one catheter was placed per 1 - 2 cm tumor diameter which resulted in a mean of 2.5 ± 1.8 catheters (range: 1 - 6 catheters) utilized per intervention depending on tumor size and configuration. After catheter positioning, either contrast enhanced multi-slice CT (collimation: 16 × 0.5 mm, slice thickness: 1 mm; table feed: 5.5 mm/rotation; 90 ml Imeron 300; flow, 3 ml/s; start delay 70 s) or T_1_-weighted fat signal saturated 3D high resolution isotropic volume examination (THRIVE; TR = 5.4 ms, TE = 2.6 ms, flip angle = 12°, section thickness of 3 mm) were acquired to depict the exact position of brachytherapy catheters in relation to tumor extension for treatment planning (Figure 1a). This was performed with the Oncentra-MasterPlan, BrachyModul planning software package (Nucleotron, Veenendaal, the Netherlands; Figure 1b). The HDR afterloading system (microSelectron Digital V3, Nucleotron, Veenendaal, the Netherlands) employed a ^192^Ir point source of 10 Ci (370 GBq). The minimal target dose prescribed for colorectal metastases was 19.4 ± 3.1 Gy (range: 10.3 - 24.0 Gy).

### MR Imaging Protocol

Magnetic resonance imaging was performed with a 1.5 T MR system (Gyroscan, Intera, Phillips Medical Systems, Best, The Netherlands) employing a SENSE torso surface coil. Imaging was performed at three time points: Baseline MRI was performed at a mean of 5 days (range: 0 - 36 days) prior to CT- or MRI-guided HDR-BT. All but one patient received early MRI one to three days after HDR-BT. Another patient was scanned five days after treatment. Follow-up MRI was performed a mean of 79 days (range: 36 - 120 days) after HDR-BT.

Unenhanced T_1_-weighted gradient echo (TR = 211 ms, TE = 5 ms, 350-mm FOV, 256 × 144 matrix, SENSE factor 2, section thickness 8 mm) and T_2_-weighted fast spin echo (TR = 1,600 ms, TE = 100 ms, flip angle = 80°, 350-mm FOV, 384 × 196 matrix, SENSE factor 2, section thickness 8 mm) axial imaging were performed before DWI and Gd-EOB-DTPA contrast medium administration.

**Figure 2:**
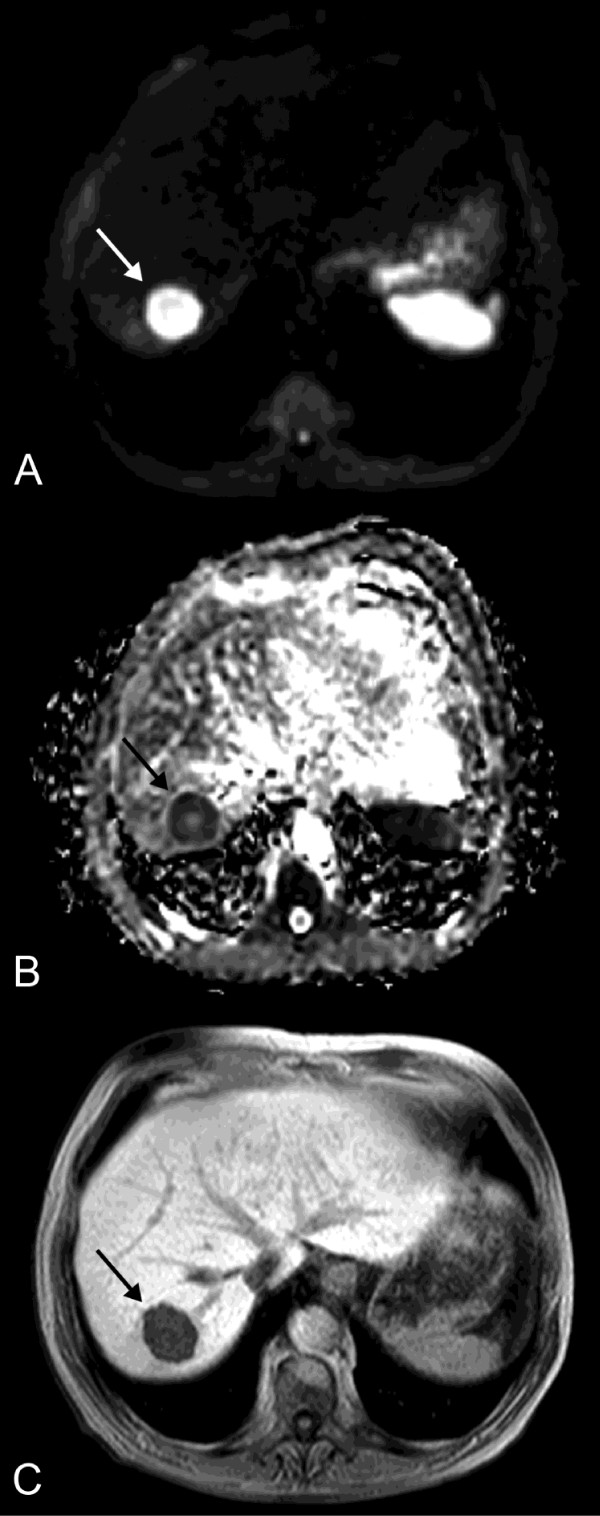
**Baseline MRI preceding HDR-BT**. Pre-treatment diffusion-weighted image (DWI) with *b *= 500 s/mm^2 ^(A), corresponding apparent diffusion coefficient (ADC) map (B) and T1w Gd-EOB-DTPA enhanced MR image in hepatocyte-selective (hepatobiliary) phase (C) of the same patient as in Figure 1 depict the colorectal metastasis in liver segment VII with a mean ADC of 1.29 × 10^-3 ^mm^2^s^-1 ^and a mean volume of 23.3 cm^3 ^(arrow).

Breath-hold axial single shot echo planar (EPI) DWI was acquired using the following parameters: TR = 1850 ms; TE = 68 ms; *b *factors 0 and 500 s/mm²; 112 × 111 matrix size, 350-mm FOV; section thickness 8 mm; NSA 2; half-scan factor 0.608. Twelve sections through the liver were acquired in each 20-s breath-hold, and the entire liver (from the level of the diaphragm to the inferior edge of the liver) was typically evaluated in two to three breath-holds (Figure 2a). ADC maps were calculated on a voxel-by-voxel basis with an implemented algorithm according to the following equation:

in which S^0 ^and S^b ^represent the signal intensities of the images with different gradient *b *factors, and *b *is the difference between gradient *b *factors (Figure 2b).

Then, 0.1 mmol/kg body weight of Gd-EOB-DTPA was administered with an infusion rate of 1.5 ml/s followed by a 30-ml saline flush. THRIVE images were acquired with the following parameters: TR = 3.9 ms, TE = 1.9 ms, flip angle = 10°, 350-mm FOV, 192 × 136 matrix, SENSE factor 2, section thickness 6 mm, spectral adiabatic inversion recovery (SPAIR). In order to minimise differences in contrast media circulation time, the first post-contrast (arterial phase) sequence was started manually by using the bolus tracking technique at the time when contrast agent reached the ascending aorta, typically 14-17 s after the start of injection. For subsequent acquisitions, intervals allowing patient's free breathing were placed between the arterial and portal venous phase (20 s) and the portal venous and equilibrium (i.e. interstitial) phase (40 s), respectively. THRIVE as well as T_1_-weighted 2D gradient echo with selective water excitation (WATS) images (TR = 131 msec, TE = 5 msec, flip angle = 70°, 350-mm FOV, 256 × 135 matrix, SENSE factor 2, section thickness 8 mm) were acquired 20 min after contrast material administration at the hepatocyte-selective (hepatobiliary) phase (Figure 2c).

### Tumor Volume Assessment and ADC Calculation

Assessment of tumor areas was performed with the OsiriX imaging software version 3.6.1. Tumor borders were segmented manually on transversal Gd-EOB-DTPA enhanced THRIVE images by two independent investigators. The mean of the volumetric measurements was taken as representative TV for each lesion. TV was expressed by OsiriX in cubic centimeters (cm^3^).

**Figure 3:**
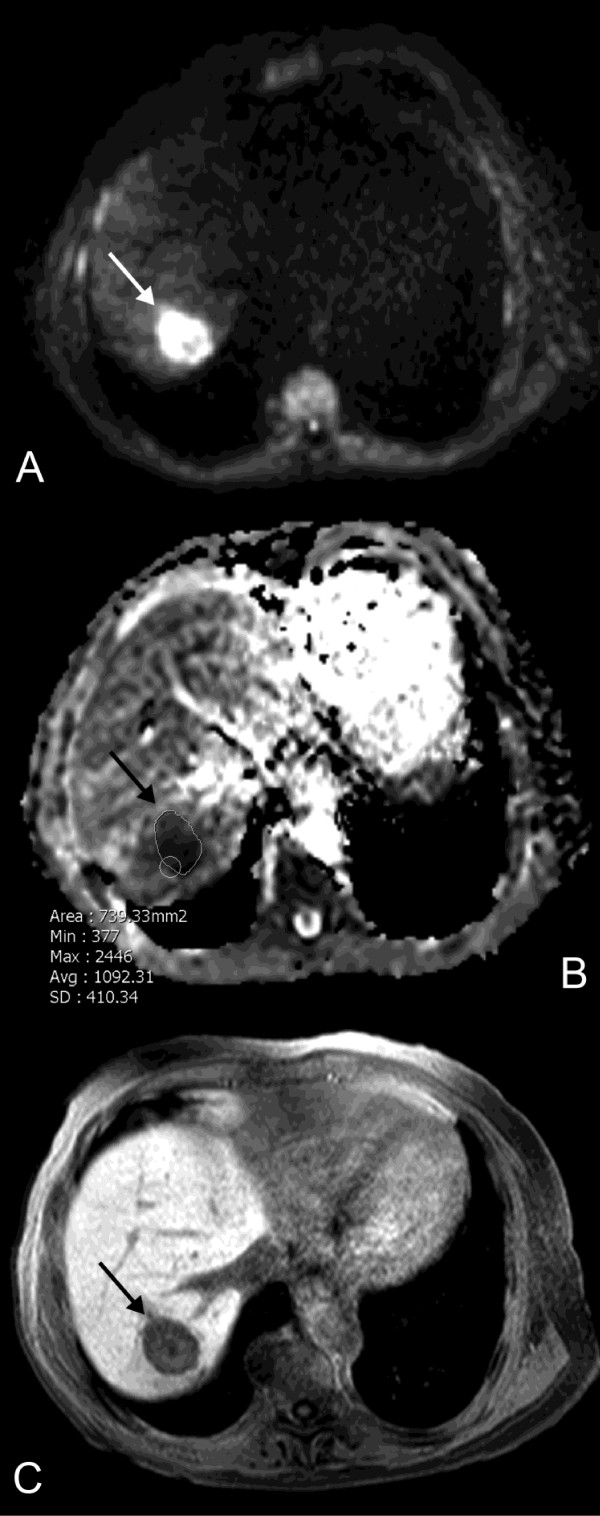
**Early MRI 3 days after HDR-BT**. Early DWI (A) and corresponding ADC map (B) performed 3 days after HDR-BT (same patient as in Figure 1) reveal a decrease in mean ADC by 27.1% to 0.94 × 10^-3 ^mm^2^s^-1^. The ROI within the lesion indicates an ADC value of 1.09 × 10^-3 ^mm^2^s^-1 ^in this slice of the ADC map (arrow). T1w Gd-EOB-DTPA enhanced MR image in hepatobiliary phase (C) indicates no relevant change in size of the treated lesion (24.1 cm^3^).

**Figure 4:**
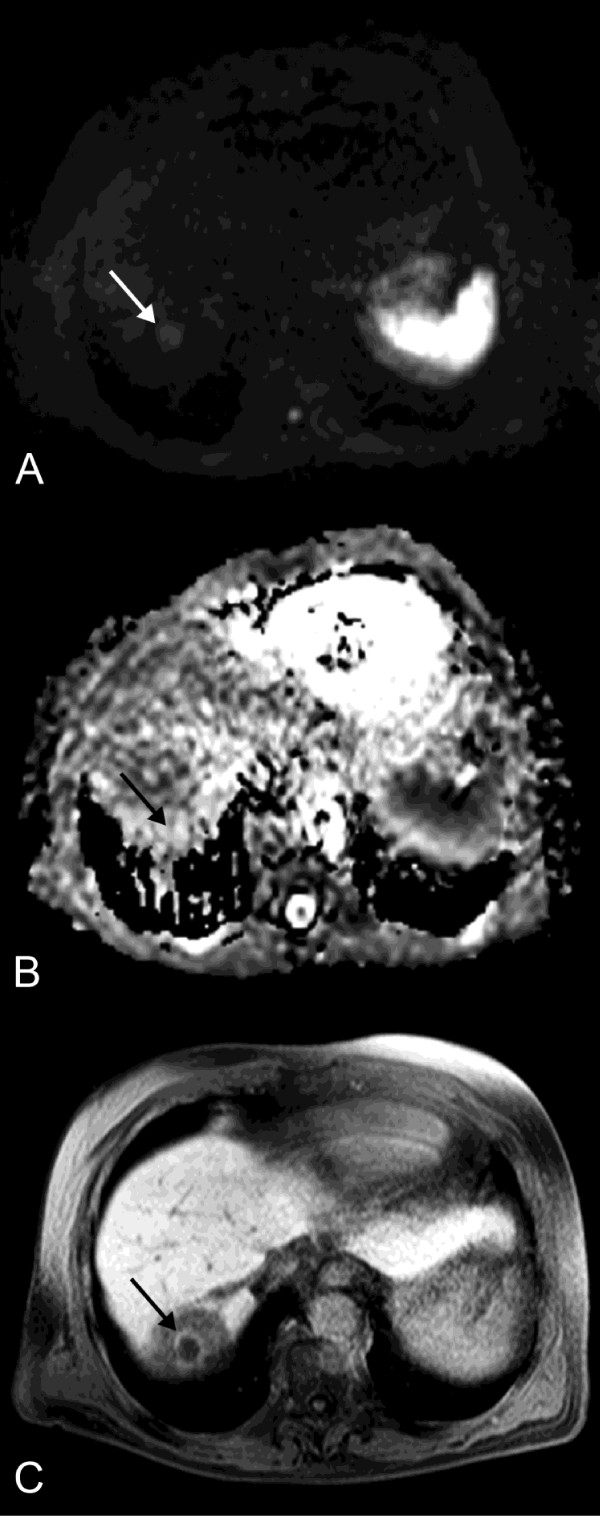
**Follow-up MRI**. DWI (A) and ADC map (B) performed 105 days post intervention (same patient as in Figure 1) show a rise of mean tumor ADC of 75.2% to 2.26 × 10^-3 ^mm^2^s^-1 ^(arrow). This finding correlates with a decrease in tumor volume by 90.6% (2.2 cm^3^), depicted in T1w Gd-EOB-DTPA enhanced MR image in hepatobiliary phase (C). The circular hypointense region around the treated lesion in (C) indicates the area of irradiation induced reversible hepatocyte dysfunction.

For ADC calculation up to three slices of the ADC map depicting the largest tumor diameter were selected, depending on the volume of the tumor. In each slice a region of interest (ROI) was delineated according to the tumor geometry. The border of the ROI was placed in the tumor periphery close to the tumor margin, so that the ROI encompassed almost the whole tumor area (Figure 3). The measurements were performed independently by two experienced investigators and the mean of the measurements was recorded as representative ADC value for each lesion. Initial and follow-up images were matched and ADC calculations were performed on corresponding sections on follow-up MRI (Figure 4).

### Statistical Analysis

SPSS, version 17.0 (Chicago, IL) was used for statistical analysis. Interobserver agreement was assessed with Cohen's Kappa (κ ≤ 0.40 poor agreement, κ = 0.41 - 075 good agreement, κ ≥ 0.76 excellent agreement).

To discuss the treatment effect, we performed a univariate comparison between treated and non-treated colorectal metastases with regards to changes in mean ADC and TV at early and follow-up MRI compared to baseline MRI using the t test (Welch test, Satterthwaite's approximation to compute the degrees of freedom).

After that we performed an ANOVA with the adjusted F-Test by Greenhouse-Geisser to get a global test for time effects in each of the two groups. Paired t test with Bonferroni correction for multiple testing was applied to test the significance of the differences of treatment induced changes of ADC values and TV between early and follow-up MRI compared to baseline MRI. The correlation between the change of the mean ADC and TV was expressed with the Pearson's correlation coefficient r. A two-tailed p-value of 0.05 was set to be the level of statistical significance.

## Results

There was an excellent interobserver agreement between the two readers with a kappa coefficient of 0.93 for the assessment of TV and 0.89 for ADC values.

At baseline, mean TV of treated colorectal liver metastases was 62.2 cm^3 ^(range: 0.5 - 786.2 cm^3^) while mean tumor ADC was 1.75 × 10^-3^mm^2^s^-1 ^(range: 0.65 - 3.22 × 10^-3^mm^2^s^-1^). In non-treated lesions mean TV was 50.0 cm^3 ^(range: 2.3 - 136.9 cm^3^) with a mean tumor ADC of 1.88 × 10^-3^mm^2^s^-1 ^(range: 1.40 - 2.67 × 10^-3^mm^2^s^-1^). The difference between treated and non-treated lesions with regards to mean TV and mean tumor ADC at baseline was non significant (*p*> 0.25).

**Figure 5:**
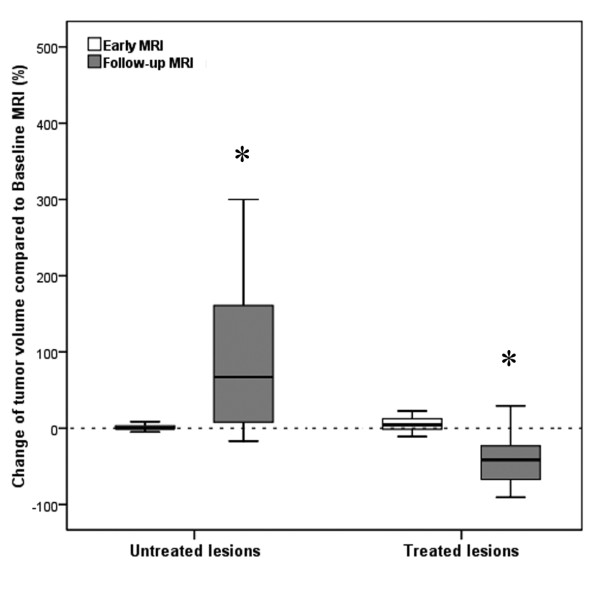
**Boxplot depicting changes of mean volume of non-treated and treated tumors at early and follow-up MRI compared to baseline MRI**. Boxplot shows changes of mean tumor volume (TV) of non-treated (*: *p *= 0.027) and treated colorectal liver metastases (*: *p *= 0.026) 2 days (early MRI) as well as 3 months (follow-up MRI) after HDR-BT as compared to baseline MRI.

**Figure 6:**
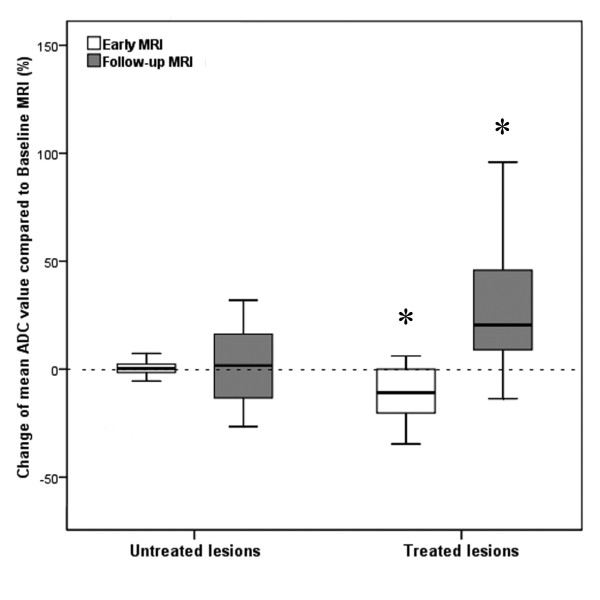
**Boxplot depicting changes of mean ADC of non-treated and treated tumors at early and follow-up MRI compared to baseline MRI**. Boxplot illustrates changes of mean ADC of non-treated and treated colorectal liver metastases 2 days (early MRI) as well as 3 months (follow-up MRI) following HDR-BT as compared to baseline MRI (*: *p *< 0.001).

The change in mean TV (*p *= 0.007) and mean tumor ADC (*p *< 0.001) differed significantly between treated and non-treated colorectal liver metastases at early MRI. No changes of TV (50.2 cm^3^; range: 2.3 - 140.6 cm^3^) as well as mean tumor ADC (1.90 × 10^-3 ^mm^2^s ^-1^; range: 1.41 - 2.64 × 10^-3^mm^2^s^-1^) were found for the non-treated lesions (Figure 5 and 6). In contrast, mean TV of colorectal liver metastases treated with HDR-BT increased by 8.8% to 67.7 cm^3 ^(range: 0.5 - 886.0 cm^3^), but only a trend towards a statistically significant difference was observed (*p *= 0.054; Figure 5). Remarkably, mean tumor ADC of treated colorectal liver metastases decreased significantly by 11.4% to 1.55 × 10^-3 ^mm^2^s ^-1 ^(range: 0.64 - 2.60 × 10^-3^mm^2^s^-1^; *p *< 0.001; Figure 6). The change between mean TV and mean tumor ADC of the treated lesions did not differ significantly between one and three days (*p *= 0.708 and *p *= 0.945).

**Figure 7:**
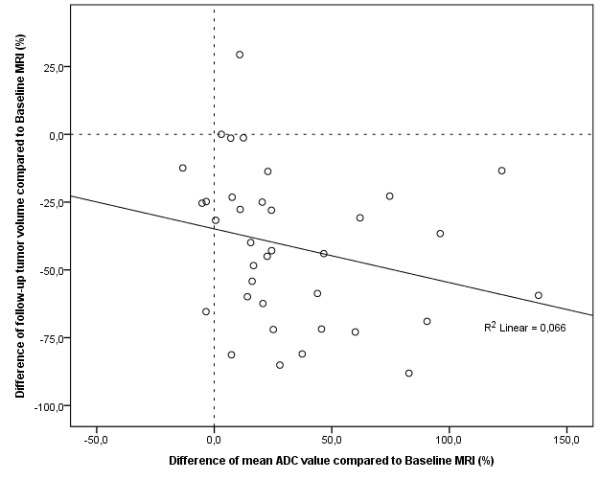
**Scatter plot depicting the relationship between changes of mean tumor volume and mean tumor ADC at follow-up MRI compared to baseline MRI**. Scatter plot depicts the relationship between changes of mean tumor volumes and mean ADC values of colorectal liver metastases 3 months after treatment with HDR-BT as compared to baseline MRI. A decrease in tumor size is inversely associated with an increase in ADC. Pearson's correlation indicated a weak but statistically significant linear relationship of *r *= -0.257 (*p *< 0.001).

The change in mean TV (*p *= 0.002) and mean tumor ADC (*p *< 0.001) differed significantly between treated and non-treated colorectal liver metastases at follow-up MRI. At follow-up MRI mean TV of non-treated colorectal liver metastases increased significantly by 50.8% to 75.4 cm^3 ^(range: 10.2 - 170.3 cm^3^) as compared to baseline (*p *= 0.027; Figure 5). Mean tumor ADC at the time of follow-up MRI was 1.92 × 10^-3 ^mm^2^s ^-1 ^(range: 1.32 - 3.23 × 10^-3^mm^2^s^-1^), which resembled a non significant change of only 1.0% (*p*> 0.9; Figure 6). In contrast, mean TV at follow-up MRI of colorectal liver metastases treated with HDR-BT decreased by 47.0% to 33.0 cm^3 ^(range: 0.5 - 397.8 cm^3^) as compared to baseline (*p *= 0.026; Figure 5). This reflected a local tumor control rate of 97.7% with absence of progression in 40 of 41 treated lesions. The mean tumor ADC increased significantly by 28.6% to 2.25 × 10^-3 ^mm^2^s ^-1 ^(range: 0.72 - 3.31 × 10^-3^mm^2^s^-1^) as compared to baseline (*p *< 0.001; Figure 6). Pearson's correlation indicated a weak but statistically significant linear relationship between the change of mean TV and mean tumor ADC of *r *= -0.257 (*p *< 0.001; Figure 7). Hence, differences in ADC were inversely correlated with morphological changes.

## Discussion

Our study demonstrated HDR-BT to be highly efficient for the treatment of unresectable colorectal liver metastases [8,10,22,23]. Furthermore, tumor size reduction was inversely correlated with a significant increase in mean tumor ADC values after 3 months. These results are well in agreement with the current understanding of therapy induced changes assessed by DWI: effective anticancer treatment results in tumor lysis, loss of cell membrane integrity, increased extracellular space, and, therefore, an increase in water diffusion [24,25]. Our results were also in accordance with results of previous studies of primary and secondary liver tumors, which all have shown an increase in ADC after a number of different therapeutic modalities [16-21].

On early MRI performed in mean 2 days after HDR-BT, DWI was able to depict tumor response as only in treated lesions mean tumor ADC values decreased significantly. A slight increase in TV accompanied the decrease in ADC (compare Figures 5 and 6). How may this *decrease *in mean tumor ADC and increase in TV be explained? Current models of tumor response postulate cell swelling to occur soon after initiation of anticancer therapy. This can lead to a transient decrease in tumor ADC [14,24,26]. Such cellular changes have been recognized as an early hallmark of cellular necrosis [27-29]. In HDR-BT applied doses in next proximity to the brachytherapy catheters can exceed 100 Gy inducing even immediate cell lysis [30,31]. Additionally, irradiation compromises tumor microvasculature by causing endothelial damage at an early stage [32]. Endothelial damage may lead to increased transient vascular permeability to macromolecules like albumin, which can become insoluble in the interstitium [33-36]. Consecutive restriction of extracellular microcirculation leads to a decrease in ADC. Restriction of the extracellular microcirculation in turn may compromise microperfusion through compression of capillaries and terminal lymph vessels [34]. As DWI provides simultaneous information on diffusion as well as microperfusion this effect may also have contributed to this early decrease in mean tumor ADC [37-39]. Cell swelling and transudation of plasma components into the extravascular-extracellular space of the tumor are also the most likely mechanisms responsible for the transient increase in TV.

Obviously, the timing of the evaluation of tumor response after the start of treatment is a key issue. For the present study, we chose to perform MRI including DWI very early at a median of 2 days following HDR-BT. Thus, we were enabled to obtain first information on the treatment response before the patient was discharged, which is routinely 2 to 3 days after HDR-BT at our institution. Although decrease in mean tumor ADC of treated colorectal liver metastases at early MRI was significant, the observed range of ADC values was relatively wide. Thus, at this early interval after HDR-BT this difference was not distinct enough to base clinical decisions in individuals exclusively on these findings. Perhaps a larger time interval of 1-2 weeks would have been superior, but we did not want to prolong hospitalization of these advanced cancer patients. Hence, larger clinical studies have to confirm the ability of DWI to identify treatment response to anticancer therapy and identify the best time point to perform early MRI, before inferences can be drawn that influence the therapeutic strategy.

## Conclusions

In conclusion, DWI is a promising imaging biomarker for early prediction of tumor response in patients with colorectal liver metastases treated with HDR-BT, yet the optimal interval between therapy and early follow-up needs to be elucidated.

## Competing interests

The authors declare that they have no competing interests.

## Authors' contributions

CW participated in the design and coordination of the study, data acquisition and analysis and drafted the manuscript. MZ and DL participated in data acquisition and analysis as well as literature review. MP, FF and JR participated in the design of the study and carried out the interventions. FWR performed the statistical analysis. GG participated in the design of the study and the treatment planning procedures. OD conceived of the study and participated in its design and coordination. All authors have read and approved the final manuscript.
